# Plastidial thioredoxin-like proteins are essential for normal embryogenesis and seed development in *Arabidopsis thaliana*

**DOI:** 10.1007/s10265-024-01611-7

**Published:** 2024-12-21

**Authors:** Yuka Fukushi, Yuichi Yokochi, Toru Hisabori, Keisuke Yoshida

**Affiliations:** 1 Laboratory for Chemistry and Life Science, Institute of Innovative Research, Tokyo Institute of Technology, Yokohama, 226-8501 Japan; 2 Present Address: The Graduate University for Advanced Studies, SOKENDAI, Shonan Village, Hayama, Kanagawa 240-0193 Japan; 3 Present Address: Laboratory for Chemistry and Life Science, Institute of Integrated Research, Institute of Science Tokyo, Yokohama, 226-8501 Japan

**Keywords:** *Arabidopsis thaliana*, Embryogenesis, Redox regulation, Seed development, Thioredoxin-like protein

## Abstract

Thiol/disulfide-based redox regulation is a key mechanism for modulating protein functions in response to changes in cellular redox status. Two thioredoxin (Trx)-like proteins [atypical Cys His-rich Trx (ACHT) and Trx-like2 (TrxL2)] have been identified as crucial for oxidizing and deactivating several chloroplast enzymes during light-to-dark transitions; however, their roles remain to be fully understood. In this study, we investigated the functions of Trx-like proteins in seed development. Using the CRISPR/Cas9 system, we generated an Arabidopsis quadruple mutant defective in ACHT1, ACHT2, TrxL2.1, and TrxL2.2 (*acht/trxl2*). This mutant showed increased seed lethality prior to maturation, with embryogenesis impaired primarily during the heart and torpedo stages, which are critical phases for plastid differentiation into chloroplasts. Using transgenic plants expressing EGFP-fused proteins, we confirmed that ACHT and TrxL2 are localized in plastids during embryogenesis. Additionally, seed development in the *acht/trxl2* mutant was further impaired under extended darkness and could not be recovered through complementation with variants of ACHT or TrxL2 lacking the redox-active Cys residue (replaced by Ser). These findings indicate that the protein-oxidation functions of ACHT and TrxL2 are important for plastid differentiation into chloroplasts, embryogenesis, and seed development.

**Supplementary Information:**

The online version contains supplementary material available at 10.1007/s10265-024-01611-7.

## Introduction

Thiol/disulfide-based redox regulation is a post-translational modification that modulates enzyme activity by reversibly altering the reduction/oxidation states of Cys residues. Thioredoxin (Trx) plays a pivotal role in redox regulation, transferring reducing power to redox-sensitive enzymes. Trx has a conserved amino acid sequence, Trp-Cys-Gly-Pro-Cys, at its active site. Using the two Cys residues, Trx catalyzes a dithiol/disulfide exchange with target enzymes, modulating their activity. Thus, Trx is essential for sensing local redox conditions and flexibly tuning cellular functions. The Trx-mediated redox regulation system is ubiquitously present in eukaryotic and prokaryotic cells (Holmgren 1985).

The redox regulation system in plant chloroplasts is unique for its coupling to the photosynthetic electron transport chain, linking it to the light (Buchanan 2016). Chloroplast Trx receives reducing power from ferredoxin (Fd), an electron carrier [2Fe-2S] protein in the electron transport chain, via Fd-Trx reductase, and then transfers it to target enzymes. Several key enzymes in photosynthesis reactions, including those in the Calvin-Benson cycle and ATP synthesis, are reductively activated by Trx (Gutle et al. 2017; Hisabori et al. 2013; Michelet et al. 2013). This system, known as the Fd/Trx pathway, enables light-dependent activation of photosynthetic functions, acknowledged as the basis of chloroplast redox regulation (Buchanan 2016). Until now, numerous chloroplast enzymes have been identified as Trx target candidates based on proteomic and biochemical studies (Yoshida and Hisabori 2023). These data raise the possibility that diverse chloroplast functions are regulated by this redox system.

Another feature of the redox regulation system in chloroplasts is the presence of multiple Trx subtypes (*f*-, *m*-, *x*-, *y*-, and *z*-types; Serrato et al. 2013). These subtypes exhibit varying redox potentials and protein surface charges, resulting in their functional divergence (e.g., distinct target selectivity; Yoshida et al. 2015; Yoshida and Hisabori 2017). Indeed, several reverse-genetic studies using *Arabidopsis thaliana* (L.) Heynh. (Arabidopsis) have shown that defects in different Trx subtypes result in different physiological traits (Naranjo et al. 2016; Okegawa and Motohashi 2015; Okegawa et al. 2023; Yoshida et al. 2015). Additionally, several proteins with Trx-like Cys-X-X-Cys sequences have been identified in chloroplasts (Chibani et al. 2021). Although the functions of these Trx-like proteins remain largely unclear, we identified one protein, Trx-like2 (TrxL2), as a protein-oxidation factor in chloroplasts (Yoshida et al. 2018). Furthermore, we found that another Trx-like protein, atypical Cys His-rich Trx (ACHT), also facilitates protein oxidation (Yokochi et al. 2019) and that TrxL2 and ACHT have different target preference for oxidation in vivo (Yokochi et al. 2021). Both TrxL2 and ACHT efficiently transfer reducing power to 2-Cys peroxiredoxin (Prx) and ultimately to hydrogen peroxide, enabling continuous oxidation of target enzymes (Yokochi et al. 2019; Yoshida et al. 2018). Thus, the biochemical properties and physiological roles of Trx-like proteins in chloroplasts have been at least partially elucidated. However, their functions in tissues/organs other than mature leaves (e.g., developing seeds) remain uncharacterized.

Previous studies have implicated the involvement of redox regulation in embryogenesis and seed development. For instance, disrupting the glutathione synthesis pathway causes an embryo-lethal phenotype in Arabidopsis (Cairns et al. 2006). An Arabidopsis mutant lacking plastidial (but not mitochondrial) glutathione reductase also exhibits a similar phenotype (Marty et al. 2019). Glutathione is the most abundant low-molecular-weight redox buffer. These studies indicate that its homeostasis in plastids is crucial for embryogenesis. Furthermore, it was recently reported that an Arabidopsis mutant lacking 2-Cys Prx displays impaired embryogenesis and seed development (Gallardo-Martinez et al. 2023). Collectively, these findings suggest some relationship between embryogenesis and the redox regulation system; however, the mechanism remains largely unclear.

As mentioned, the two Trx-like proteins ACHT and TrxL2 work in concert with 2-Cys Prx in the protein-oxidation pathway (Yoshida and Hisabori 2023). It has also been reported that ACHT and TrxL2 can receive reducing power from glutathione, at least in vitro (Chibani et al. 2012; Yoshida et al. 2018). These findings prompted our consideration of Trx-like protein involvement in embryogenesis and seed development. Specifically, we aimed to elucidate the roles of ACHT and TrxL2 in these processes through a reverse-genetic approach. To this end, we generated a quadruple mutant deficient in ACHT1, ACHT2, TrxL2.1, and TrxL2.2 in Arabidopsis. We characterized embryogenesis and seed development patterns in this mutant, concluding that the protein-oxidation functions of ACHT and TrxL2 are important for these processes.

## Materials and methods

### Plant materials and growth conditions

Arabidopsis ecotype Columbia-0 was used as the wild-type plant. Plants were grown in soil in a controlled growth chamber under long-day conditions (60–70 μmol photons m^−2^ s^−1^, 22 °C, and a 16/8-h day/night cycle). Plants grown for 5–7 weeks were used for analyses of embryogenesis and seed development. For the continuous darkness treatment, flower parts were covered with aluminum foil to block light [at 0 days after flowering (DAF)]. For the continuous light treatment, plants were grown under constant light for 5–6 weeks (60–70 μmol photons m^−2^ s^−1^ at 22 °C).

### CRISPR/Cas9-based gene editing

The Arabidopsis double mutant deficient in ACHT1 and ACHT2 (*acht*) and single mutant deficient in TrxL2.1 were generated using the CRISPR/Cas9 system in a previous study (Yokochi et al. 2021). In the present study, a double mutant deficient in TrxL2.1 and TrxL2.2 (*trxl2*) was generated by introducing the CRISPR/Cas9-based mutation in the *TrxL2.2* gene within the TrxL2.1-deficient background. CRISPR/Cas9-based gene editing was performed following the methods of Hahn et al. (2017), with mutation sites shown in Fig. S1. The quadruple *acht/trxl2* mutant was generated by crossing the *acht* and *trxl2* mutants and screening for homozygous quadruple mutants based on DNA sequencing analysis. Primers used for mutant screening are shown in Table S1.

### Expression of EGFP-fused ACHT and TrxL2 in Arabidopsis

The full-length *ACHT1* (*At4g26160*), *ACHT2* (*At4g29670*), *TrxL2.1* (*At5g06690*), and *TrxL2.2* (*At5g04260*) genes were cloned into the pRI201-AN vector (Takara). The EGFP-coding sequence was fused to the C-terminal end of each gene, using a linker sequence encoding Gly-Gly-Gly-Gly-Gly-Ala (Tian et al. 2004). Arabidopsis wild-type plants were transformed with the resulting plasmid via the *Agrobacterium*-mediated floral dip method (Clough and Bent 1998). Screening was conducted based on antibiotic resistance and EGFP fluorescence. Primers used for constructing transgenic plants are shown in Table S1.

### Complementation of ACHT and TrxL2 Cys-to-Ser variants in *acht/trxl2* background

Full-length *ACHT1* and *TrxL2.1* genes were cloned into the pRI201-AN vector as described above. The Cys-to-Ser mutations were introduced using the PrimeSTAR Mutagenesis Basal Kit (Takara) following the manufacturer’s instructions (Cys^128^ to Ser in ACHT1; Cys^133^ to Ser in TrxL2.1). Arabidopsis *acht/trxl2* mutant plants were transformed with the resulting plasmid via the *Agrobacterium*-mediated floral dip method (Clough and Bent 1998). Screening was conducted based on antibiotic resistance and western blot analysis using antibodies against ACHT1 and TrxL2.1 (Yokochi et al. 2021; Yoshida et al. 2018).

### Seed development and embryogenesis analyses

Seed development and embryogenesis were analyzed following the method of Feng and Ma (2017). Siliques were harvested at the indicated DAF, opened under a stereomicroscope, and seed morphology was observed. For embryo analysis, ovules were dissected from seeds under a stereomicroscope and incubated in Hoyer’s solution [20:1:6 (w/v/v) chloral hydrate:glycerol:water] for clearing (Meinke et al. 1994). After 2 days of incubation at room temperature in the dark, embryo images were obtained using differential interference contrast microscopy.

### Confocal fluorescence microscopy

During embryogenesis, EGFP-derived signals were analyzed using confocal fluorescence microscopy (Ti-E with Yokogawa CSU-W1 spinning disk, Nikon). Embryos were isolated from ovules harvested at various developmental stages and placed on glass slides with 6% (v/v) glycerol (Feng and Ma 2017). EGFP fluorescence was observed with excitation and emission at 470 and 490–550 nm, respectively. Chlorophyll autofluorescence was observed with excitation and emission at 640 and 665–715 nm, respectively.

## Results and discussion

### *acht/trxl2* mutant exhibits impaired seed development

In a previous study, we generated an Arabidopsis double mutant deficient in ACHT1 and ACHT2 (*acht* mutant) and a single mutant deficient in TrxL2.1, using the CRISPR/Cas9 system (Yokochi et al. 2021). In these mutants, each corresponding gene had a 1-bp insertion at the mutation site, resulting in frameshift mutations (Fig. S1). In the present study, we generated a double mutant deficient in TrxL2.1 and TrxL2.2 (*trxl2* mutant). Furthermore, we generated a quadruple mutant deficient in ACHT1, ACHT2, TrxL2.1, and TrxL2.2 (*acht/trxl2* mutant) by crossing the *acht* and *trxl2* mutants (Fig. S1). The *acht*, *trxl2*, and *acht/trxl2* mutants displayed no visible change in rosette leaf development under standard long-day growth conditions (Fig. S2).

**Fig. 1:**
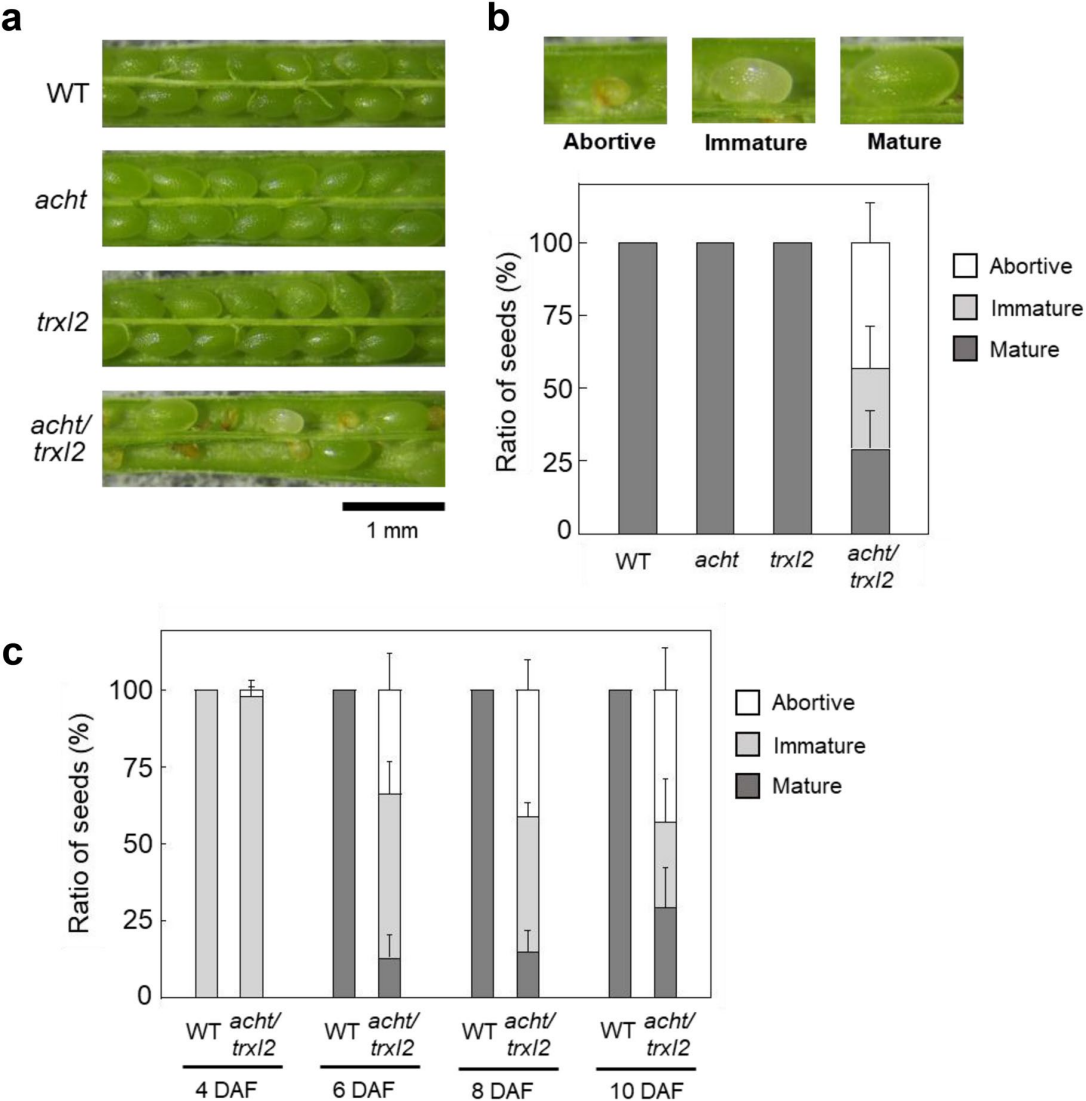
Seed development in the Arabidopsis wild-type (WT) plant, the *acht* mutant, the *trxl2* mutant, and the *acht/trxl2* mutant. **a** Microscopic images of seeds obtained at 10–11 DAF. **b** Ratios of abortive, immature, and mature seeds at 10–11 DAF. **c** Developmental changes in the ratios of abortive, immature, and mature seeds in the WT plant and the *acht/trxl2* mutant, analyzed at 4, 6, 8, and 10 DAF. Ratios were calculated as the number of seeds in each category divided by the total in one silique. Data are presented as means ± SD (*n* = 3–8)

We examined seed morphology at 10–11 DAF. All seeds in wild-type, *acht* mutant, and *trxl2* mutant plants were mature and green, whereas the *acht/trxl2* mutant contained abortive brownish seeds and immature white seeds (Fig. 1a, b). Comparing the seed morphology transitions between the wild-type and *acht/trxl2* mutant plants, we found that wild-type seeds matured by 6 DAF, whereas a significant proportion of seeds in the *acht/trxl2* mutant was abortive or immature during 6–10 DAF (Fig. 1c). These results indicate that ACHT and TrxL2 are cooperatively involved in seed development.

### *acht/trxl2* mutant shows impaired embryogenesis

**Fig. 2:**
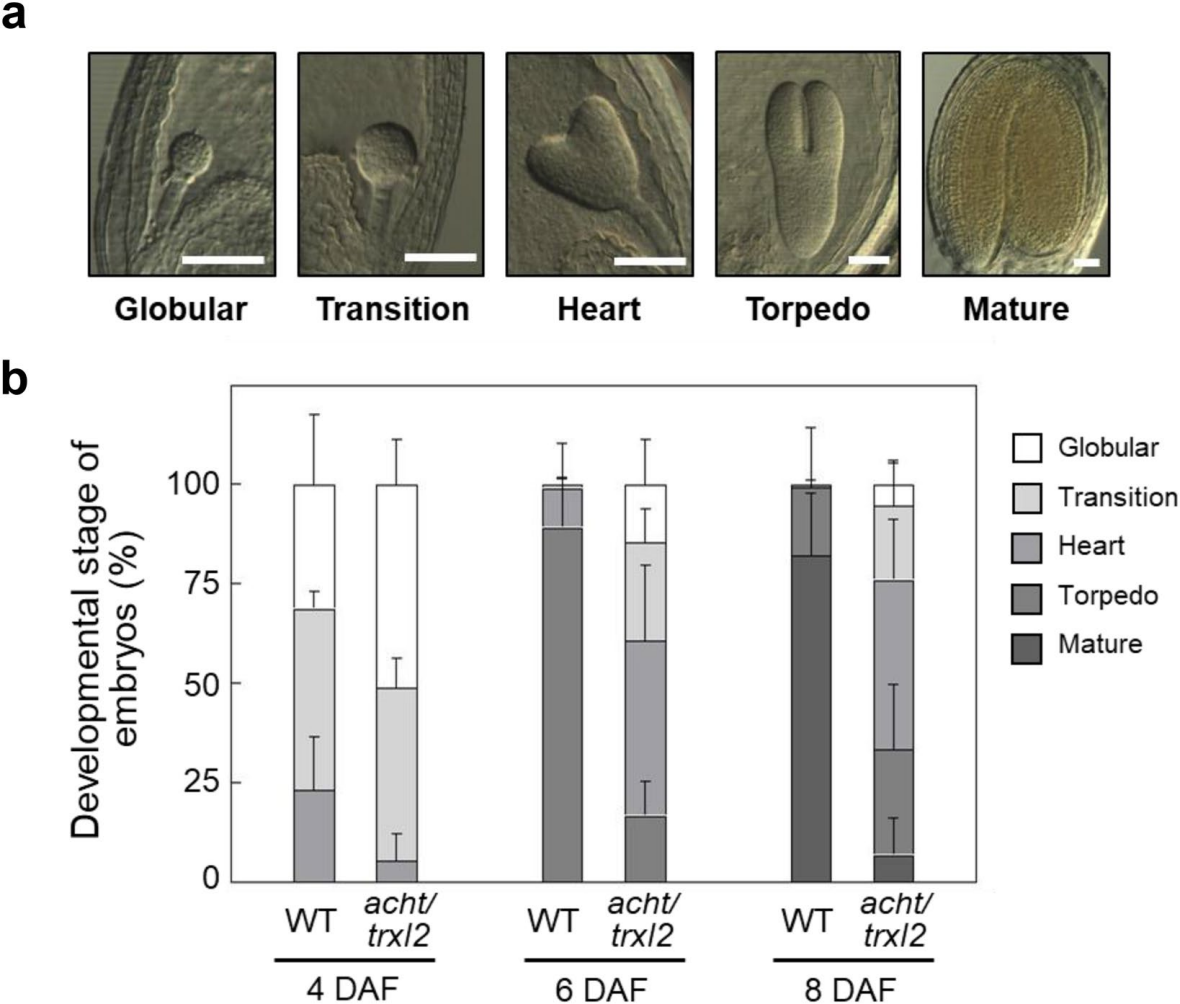
Embryogenesis in the Arabidopsis wild-type (WT) plant and the *acht/trxl2* mutant. **a** Images of embryos at the globular, transition, heart, torpedo, and mature stages. Scale bars: 50 μm. **b** Developmental changes in ratios of embryo stages, analyzed at 4, 6, 8, and 10 DAF. Ratios were calculated as the number of embryos at each stage divided by the total in one silique. Data are presented as means ± SD (*n* = 3–6)

In Arabidopsis, embryogenesis progresses through the globular, transition, heart, and torpedo stages, ultimately reaching maturity (Armenta-Medina et al. 2021). We compared the embryogenic patterns of the wild-type and *acht/trxl2* mutant plants, counting the number of embryos in each developmental stage at 4, 6, and 8 DAF (Fig. 2). In the wild-type plant, over 80% of embryos were in the torpedo and mature stages at 6 and 8 DAF, respectively. In contrast, embryogenesis in the *acht/trxl2* mutant was severely impaired; embryos in the heart stage were still dominant even at 8 DAF, with less than 10% of embryos in the mature stage. Thus, impaired seed development observed in the *acht/trxl2* mutant appears to be associated with impaired embryogenesis.

### ACHT and TrxL2 are present in plastids during embryogenesis

**Fig. 3:**
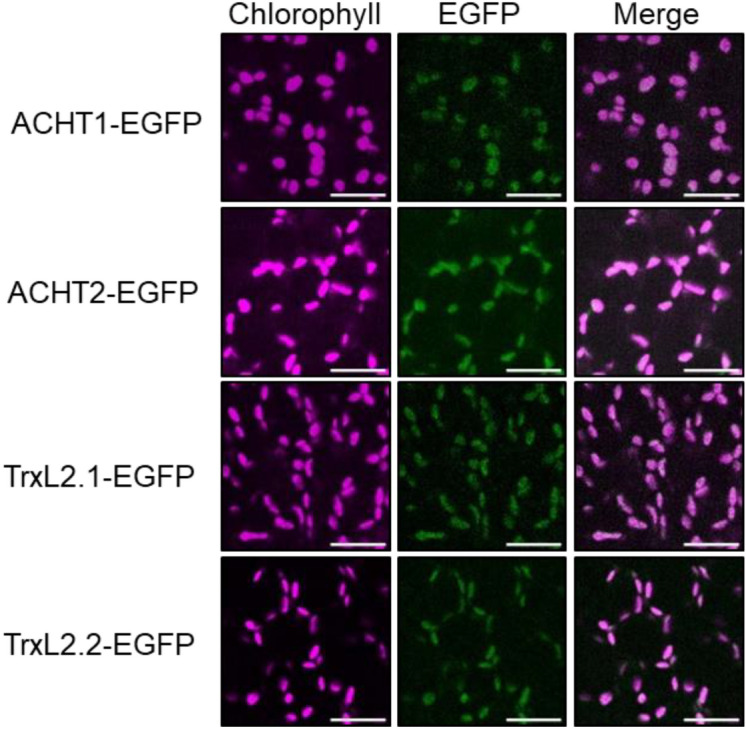
Intracellular localization of ACHT1, ACHT2, TrxL2.1, and TrxL2.2 in mature embryos. Representative images of chlorophyll autofluorescence and EGFP fluorescence are shown, along with merged images. Scale bars: 10 μm

ACHT and TrxL2 were shown to be targeted to the chloroplast in Arabidopsis leaves (Dangoor et al. 2009; Yoshida et al. 2018), but their localizations during embryogenesis remain unclear. The Arabidopsis transcriptomic database (The Arabidopsis eFP Browser; http://bar.utoronto.ca/efp/cgi-bin/efpWeb.cgi) indicates that the *ACHT1*, *ACHT2*, *TrxL2.1*, and *TrxL2.2* genes are all expressed, although at different levels, during embryogenesis (Fig. S3). To examine protein localization during embryogenesis, we constructed transgenic plants expressing EGFP fused to the ACHT1, ACHT2, TrxL2.1, and TrxL2.2 proteins under the control of the cauliflower mosaic virus *35S* promoter. Confocal fluorescence microscopy analyses showed that all four proteins were stably detected throughout embryogenesis (Fig. S4), although our observation did not reflect the endogenous expression pattern. Chlorophyll autofluorescence began to occur in the torpedo stage and increased in the mature stage, aligning with chloroplast differentiation, including chlorophyll accumulation and thylakoid membrane formation, during the heart and torpedo stages (Mansfield and Briarty 1991). We assessed the intracellular localization of the four proteins in the mature stage, with each of their signals clearly overlapping with chlorophyll autofluorescence (Fig. 3). These results suggest that ACHT and TrxL2 play key roles in plastid differentiation into chloroplasts during embryogenesis.

### Impairment of seed development in the *acht/trxl2* mutant is more pronounced under extended darkness

**Fig. 4:**
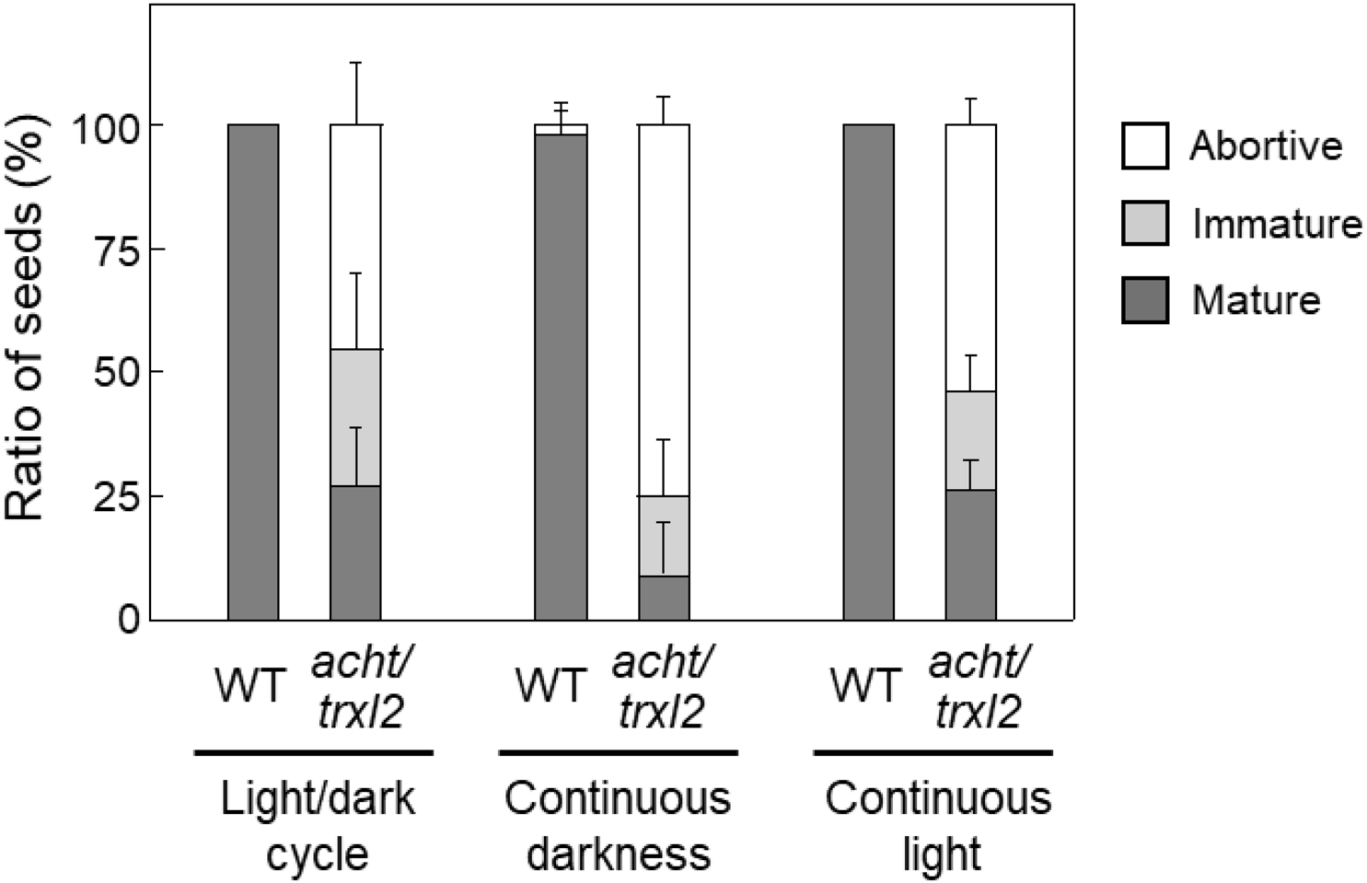
Effects of light conditions on seed development in the Arabidopsis wild-type (WT) and *acht/trxl2* mutant plants. The ratios of abortive, immature, and mature seeds at 10–11 DAF are shown. Data are presented as means ± SD (*n* = 3–8)

We investigated whether light conditions affect seed development (Fig. 4). Seed development in the wild-type plant was minimally affected even under continuous darkness, with nearly all seeds reaching maturity, similar to control conditions (day/night cycle). Conversely, seed development in the *acht/trxl2* mutant worsened under continuous darkness, with over 90% of seeds being abortive or immature. When exposed to continuous light, seed development patterns remained largely unchanged in both the wild-type and *acht/trxl2* mutant plants. These results indicate that the roles of ACHT and TrxL2 in seed development are more important during prolonged dark periods.

### Cys-to-Ser variant forms of ACHT and TrxL2 fail to recover the seed development phenotype in the *acht/trxl2* mutant

**Fig. 5:**
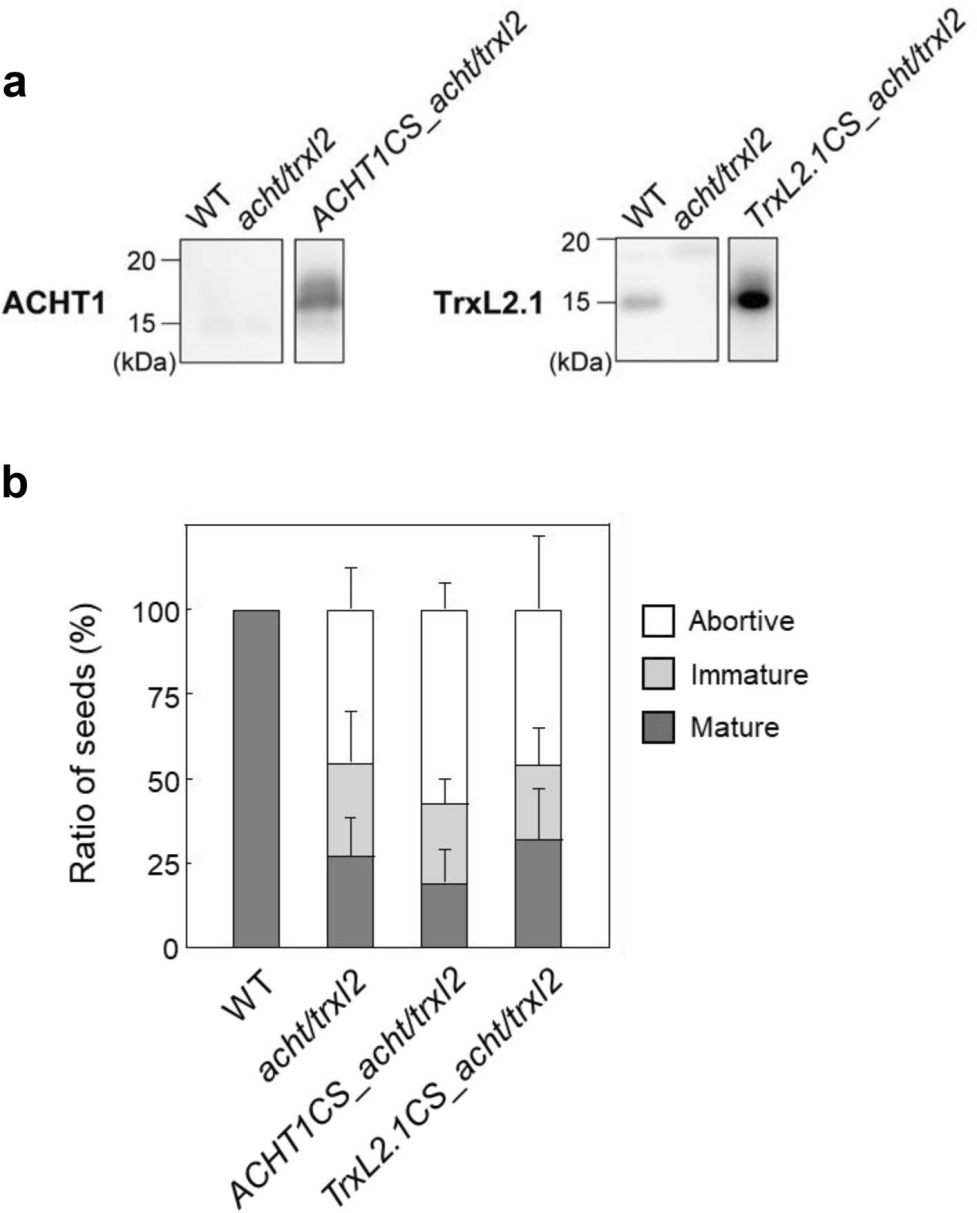
Effects of complementing ACHT1 or TrxL2.1 Cys-to-Ser variants in the *acht/trxl2* background (*ACHT1CS_acht/trxl2* or *TrxL2.1CS_acht/trxl2*, respectively) on seed development. **a** Western blot analyses of ACHT1 and TrxL2.1. The same amount of total leaf protein was loaded in each lane. The ACHT1 expression level was below the detection limit even in the wild-type plant (Yokochi et al. 2021). **b** Ratios of abortive, immature, and mature seeds at 10–11 DAF. Data are presented as means ± SD (*n* = 3–8)

To determine whether the Cys residue in the active sites of ACHT and TrxL2 is essential for seed development, we complemented the variant forms of ACHT or TrxL2 in the *acht/trxl2* mutant background. In these variants, the Cys-X-X-Cys sequence in each active site was replaced with Cys-X-X-Ser, which is thought to interfere with the dithiol-disulfide exchange reaction, although TrxL2 may reduce some enzymes in a monothiol manner (Chibani et al. 2012). Western blot analyses indicated that both the ACHT1 and TrxL2.1 variant forms were more abundantly expressed in the complemented strains than in the wild-type plants (Fig. 5a). Nevertheless, the seed development patterns of these complemented strains were nearly identical to those of the *acht/trxl2* mutant (Fig. 5b). We also investigated seed development in plants in which only ACHT1 or TrxL2.1 is functional among the four Trx-like proteins (the *acht2/trxl2.1/trxl2.2* or *acht1/acht2/trxl2.2* triple mutant, respectively; Fig. S5). In these mutants, nearly all seeds reached maturity, as in the wild-type plant. These results indicate that the active site sequence of ACHT or TrxL2 is required for normal seed development. As mentioned, ACHT and TrxL2 have been shown to efficiently oxidize target enzymes (Yokochi et al. 2021, 2019; Yoshida et al. 2018). Therefore, we suggest that the protein-oxidation functions of ACHT and TrxL2 are involved in seed development.

Given that photosynthesis occurs even in developing seeds (Allorent et al. 2015; Puthur et al. 2013), plastidial redox-sensitive enzymes in these tissues are potentially reduced in response to light. If so, the oxidation of these enzymes, mediated by ACHT and TrxL2, is thought to be important for balancing their activities and finely tuning plastidial functions under changing light conditions. This may explain the more pronounced impairment of seed development in the *acht/trxl2* mutant under extended darkness (Fig. 4), as redox-sensitive enzymes must be stably present in an oxidized state under such conditions. This issue should be tested experimentally in the future by determining protein redox dynamics during seed development.

### Involvement of ACHT and TrxL2 in embryogenesis and seed development

Although we provide evidence for the novel roles of ACHT and TrxL2 in embryogenesis and seed development, an important question remains: which enzymes are oxidation targets of ACHT and TrxL2 in embryo plastids? One candidate is glucose-6-phosphate dehydrogenase (G6PDH), involved in the plastidial oxidative pentose phosphate pathway (OPPP; Kruger and von Schaewen 2003). The OPPP is essential for embryogenesis, supplying intermediates and reducing power for several biosynthetic pathways in plastids (e.g. nucleotide synthesis; Andriotis and Smith 2019). Actually, Arabidopsis mutants lacking certain OPPP enzymes exhibit arrested embryogenesis (Andriotis and Smith 2019). A similar phenotype occurs in Arabidopsis mutants lacking the plastidial glucose-6-phosphate/phosphate antiporter, necessary for importing OPPP substrates (Andriotis et al. 2010). The first committed reaction of the OPPP is catalyzed by G6PDH, a well-known target of redox regulation. Importantly, unlike most redox-sensitive enzymes (e.g. Calvin-Benson cycle enzymes), G6PDH is active in the oxidized state (Scheibe and Anderson 1981; Scheibe et al. 1989). Thus, ACHT and TrxL2 may play critical roles in cooperatively oxidizing and activating G6PDH, thereby maintaining OPPP activity during embryogenesis. This notion is supported by the substantial expression of redox-sensitive G6PDH isoform in Arabidopsis siliques (Wakao and Benning 2005). Furthermore, our in vitro analysis demonstrated that TrxL2 serves as an oxidative activator of G6PDH (Yoshida et al. 2019), providing a biochemical foundation for the proposed process.

In Arabidopsis, many genes essential for embryogenesis are categorized as *EMBRYO-DEFECTIVE* (*EMB*) genes (https://seedgenes.org/; Meinke 2020). Such genes encode hundreds of proteins that catalyze a wide range of biological processes. It is plausible that ACHT and TrxL2 interact with certain *EMB* gene products, which is vital for proper embryogenesis. Further studies are warranted to identify proteins that are oxidatively regulated by ACHT and TrxL2 in embryo plastids.

## Conclusions

This study reports the functional requirement of ACHT and TrxL2 in normal embryogenesis and seed development. A notable finding is the heterogeneous seed phenotypes, including abortive, immature, and mature seeds, in the *acht/trxl2* mutant (Fig. 1). The mechanism for this heterogeneity is unclear at this stage, but may be related to the uneven microenvironment within a silique. Importantly, these heterogeneous seed phenotypes are quite similar to those in mutants lacking 2-Cys Prx (Gallardo-Martinez et al. 2023). Therefore, as in leaf chloroplasts, ACHT and TrxL2 may form protein-oxidation pathways alongside 2-Cys Prx in embryo plastids. In addition, unique embryonic photosynthesis (Allorent et al. 2015) may need specific roles of such protein-oxidation pathways. Our findings provide a foundation for further research elucidating the novel functions of Trx-like proteins.

## Supplementary Information

Below is the link to the electronic supplementary material.

Supplementary file1 (PDF 671 KB)

## Data Availability

The data that support the findings of this study are available from the corresponding author upon reasonable request.
