# Supplementary material for: Plastidial thioredoxin-like proteins are essential for normal embryogenesis and seed development in Arabidopsis thaliana
Source: J Plant Res. 2024 Dec 21;138(2):337–45. doi: 10.1007/s10265-024-01611-7 (PMC11910432; doi:10.1007/s10265-024-01611-7)
Supplement: Supplementary file 1 — Supplementary file1 (PDF 671 KB) [file 10265_2024_1611_MOESM1_ESM.pdf]

Electronic supplementary materials

Title:

Plastidial thioredoxin-like proteins are essential for normal embryogenesis and seed development in *Arabidopsis thaliana*

Authors:

Yuka Fukushi, Yuichi Yokochi, Toru Hisabori, Keisuke Yoshida

Journal:

Journal of Plant Research

Corresponding author:

Keisuke Yoshida

(Laboratory for Chemistry and Life Science, Institute of Integrated Research, Institute of Science Tokyo, Yokohama 226-8501 Japan)

Tel: +81-45-924-5859

Fax: +81-45-924-5859

E-mail: kyoshida@res.titech.ac.jp

Content:

Table S1

Figs. S1–S5

Table S1. Primers used in this study

Name	Sequence (5' to 3')	Purpose
ACHT1_F	CTTCTCGCTTTACGCTTCTTC	Screening for mutant plants
ACHT2_F	CAAGTGGATGATGGCGG	Screening for mutant plants
TrxL2.1_F	ACTTAAAAATATCTCGTCAC	Screening for mutant plants
TrxL2.2_F	TTCTAGTAGCAACACGAATCCG	Screening for mutant plants
Trxlike_vector_F	TACAAACTCGAGTAATCGACAATTCTGAATCAACAACTC	Constructing transgenic plants expressing EGFP-fused Trx-like proteins
Trxlike_insert_F	GGAGGTGGAGGTGGAGCTGCTAGCAAAGGAGAAGAACTC	Constructing transgenic plants expressing EGFP-fused Trx-like proteins
Trxlike_insert_R	GAATTGTCGATTACTCGAGTTTGTATAGTTCATCC	Constructing transgenic plants expressing EGFP-fused Trx-like proteins
ACHT1_vector_R	TGCTAGCAGCTCCACCTCCACCTCCTTCACTTGAATCTTCAACTTTCTC	Constructing transgenic plants expressing EGFP-fused Trx-like proteins
ACHT2_vector_R	TGCTAGCAGCTCCACCTCCACCTCCACTTGATGCAGCTGGTTTGG	Constructing transgenic plants expressing EGFP-fused Trx-like proteins
TrxL2.1_vector_R	TGCTAGCAGCTCCACCTCCACCTCCGACAAATTTGTTGATCAATTCTC	Constructing transgenic plants expressing EGFP-fused Trx-like proteins
TrxL2.2_vector_R	TGCTAGCAGCTCCACCTCCACCTCCAGTGATTGAATCGTTCTCTATC	Constructing transgenic plants expressing EGFP-fused Trx-like proteins

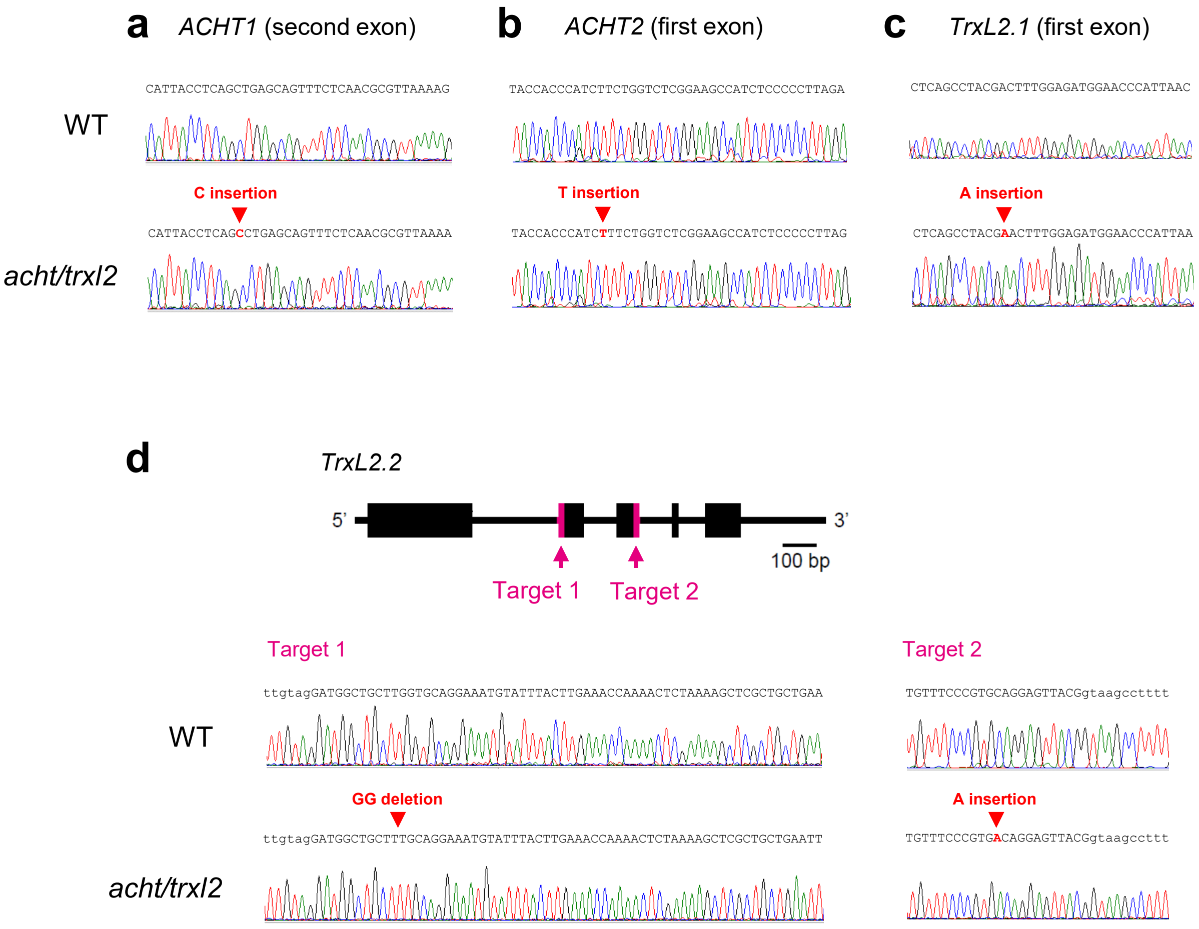

Fig. S1 DNA sequences surrounding the Cas9-targeted site in the *Arabidopsis* wild-type (WT) and *acht/trxl2* mutant plants. Simplified gene structures for *ACHT1* (a), *ACHT2* (b), and *TrxL2.1* (c) are described in a previous study (Yokochi et al. 2021). The simplified gene structure for *TrxL2.2* and two Cas9-targeted sites in this gene are shown in (d).

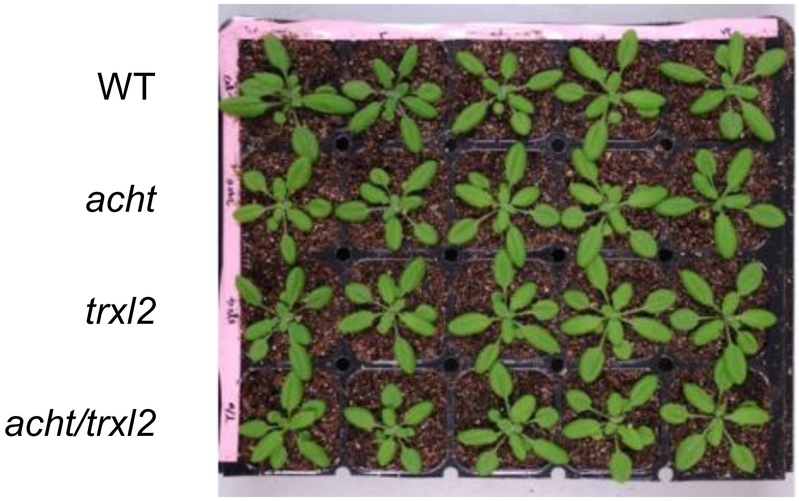

Fig. S2 Visible phenotypes of rosette leaf development in the *Arabidopsis* wild-type (WT) plant, the *acht* mutant, the *trxl2* mutant, and the *acht/trxl2* mutant grown under long-day conditions for 4 weeks.

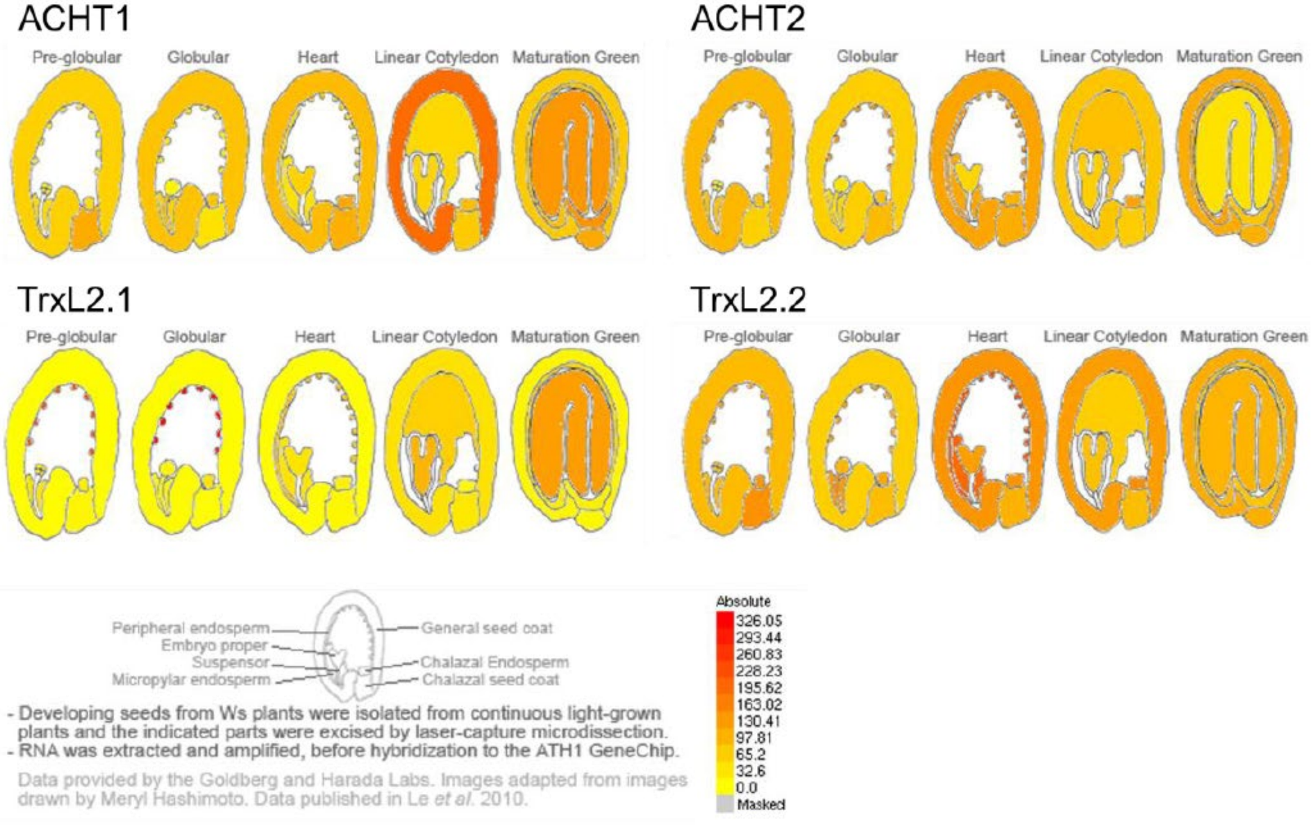

Fig. S3 Expression patterns of *ACHT1*, *ACHT2*, *TrxL2.1*, and *TrxL2.2* genes during seed development. Data were collected from the Arabidopsis eFP browser (http://bar.utoronto.ca/efp/cgi-bin/efpWeb.cgi).

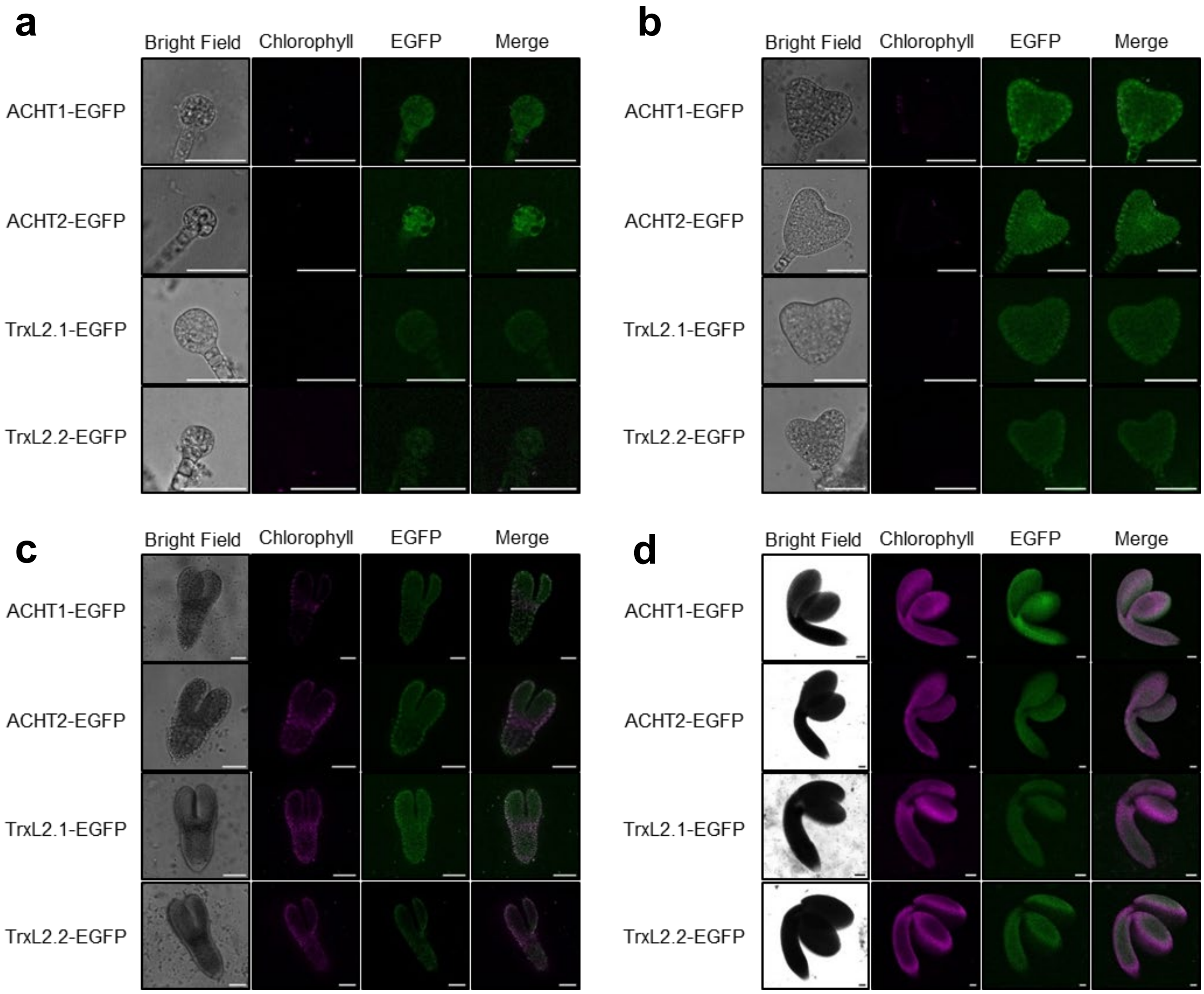

Fig. S4 Distribution of ACHT1, ACHT2, TrxL2.1, and TrxL2.2 proteins during embryogenesis. Representative images of bright field, chlorophyll autofluorescence, and EGFP fluorescence are shown, along with merged images of chlorophyll autofluorescence and EGFP fluorescence. Analyses were performed at the globular (a), heart (b), torpedo (c), and mature stages (d). Scale bars: 50 μm.

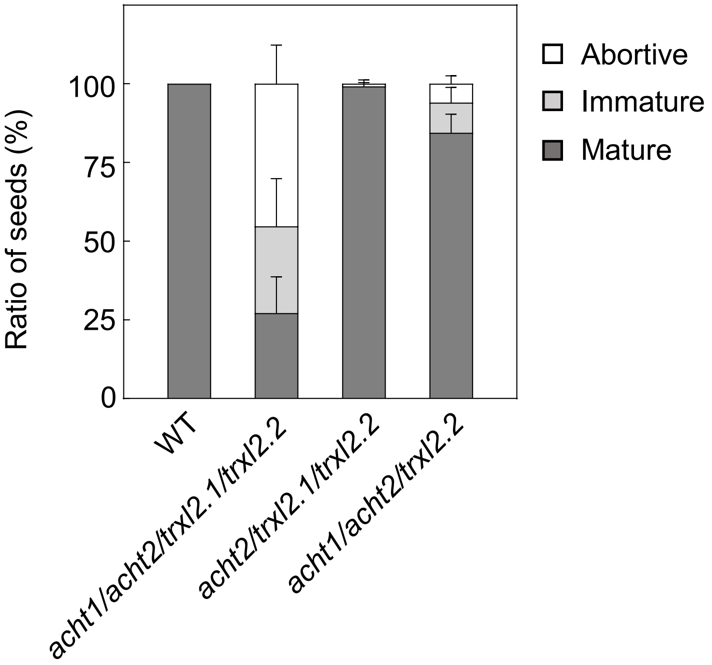

Fig. S5 Seed development in the *Arabidopsis* wild-type (WT) plant, the *acht1/acht2/trxl2.1/trxl2.2* quadruple mutant (described as the *acht/trxl2* mutant elsewhere), the *acht2/trxl2.1/trxl2.2* triple mutant, and *acht1/acht2/trxl2.2* triple mutant. Ratios of abortive, immature, and mature seeds at 10–11 DAF are shown. Data are presented as means ± SD (n = 3–8).
